# Human Lysozyme Synergistically Enhances Bactericidal Dynamics and Lowers the Resistant Mutant Prevention Concentration for Metronidazole to *Helicobacter pylori* by Increasing Cell Permeability

**DOI:** 10.3390/molecules21111435

**Published:** 2016-10-28

**Authors:** Xiaolin Zhang, Anmin Jiang, Hao Yu, Youyi Xiong, Guoliang Zhou, Meisong Qin, Jinfeng Dou, Jianfei Wang

**Affiliations:** 1 The Department of Pharmacy, Food and Drug School, Anhui Science and Technology University, Fengyang 233100, China; yuh@ahstu.edu.cn (H.Y.); xiongyy@ahstu.edu.cn (Y.X.); zhougl@ahstu.edu.cn (G.Z.); qinms@ahstu.edu.cn (M.Q.); doujf@ahstu.edu.cn (J.D.); 2 The School of Life Science, University of Science and Technology of China, Hefei 230032, China; SA12226011@mail.ustu.edu.cn; 3 The Ministry of Agriculture Key Laboratory of Microbial Organic Fertilizer, Bengbu 233030, China

**Keywords:** *H. pylori*, metronidazole, human lysozyme, mutant prevention concentration, bactericidal dynamics

## Abstract

Metronidazole (MNZ) is an effective agent that has been employed to eradicate *Helicobacter pylori* (*H. pylori*). The emergence of broad MNZ resistance in *H. pylori* has affected the efficacy of this therapeutic agent. The concentration of MNZ, especially the mutant prevention concentration (MPC), plays an important role in selecting or enriching resistant mutants and regulating therapeutic effects. A strategy to reduce the MPC that can not only effectively treat *H. pylori* but also prevent resistance mutations is needed. *H. pylori* is highly resistant to lysozyme. Lysozyme possesses a hydrolytic bacterial cell wall peptidoglycan and a cationic dependent mode. These effects can increase the permeability of bacterial cells and promote antibiotic absorption into bacterial cells. In this study, human lysozyme (hLYS) was used to probe its effects on the integrity of the *H. pylori* outer and inner membranes using as fluorescent probe hydrophobic 1-*N*-phenyl-naphthylamine (NPN) and the release of aspartate aminotransferase. Further studies using a propidium iodide staining method assessed whether hLYS could increase cell permeability and promote cell absorption. Finally, we determined the effects of hLYS on the bactericidal dynamics and MPC of MNZ in *H. pylori*. Our findings indicate that hLYS could dramatically increase cell permeability, reduce the MPC of MNZ for *H. pylori*, and enhance its bactericidal dynamic activity, demonstrating that hLYS could reduce the probability of MNZ inducing resistance mutations.

## 1. Introduction

*H. pylori* is a widespread human pathogen that colonizes over half of all individuals worldwide and up to 90% of people in some developing countries [1]. The stomachs of many individuals are infected by *H. pylori*, usually during childhood. *H. pylori* plays a decisive role in the development of peptic ulcers and chronic gastritis [2,3]. *H. pylori* is a carcinogen that is closely associated with the occurrence of gastric carcinoma and the development of mucosa-associated lymphoid tissue (MALT) lymphoma [4]. It has been established that the eradication of *H. pylori* would be the best strategy to prevent the gastrointestinal disorders of gastroduodenal ulcer and chronic gastritis [2,3,5,6] and to reduce the incidence of gastric cancer [3,7]. Moreover, some reports indicate that the eradication of *H. pylori* would be beneficial for treatment of vitamin B12 deficiency, idiopathic thrombocytopenic purpura and unexplained iron deficiency anemia [8]. Additionally, it could reduce the emergence of ischemic heart disease, colorectal cancer and neurological disorders [9,10]. MNZ is an effective agent that can be used to eradicate *H. pylori*. However, the emergence of widespread MNZ resistance has affected the therapeutic outcome of *H. pylori* infections. It has been reported that resistance to MNZ may cause the cure efficiency using standard triple therapies to decrease by up to 50% [11]. To overcome *H. pylori* resistance, an increasing MNZ dose and an extended period of therapy may be used [12]. However, it has become evident that treatment using high concentrations of antibiotics can enhance the selection of resistant mutants [13,14].

**Figure 1:**
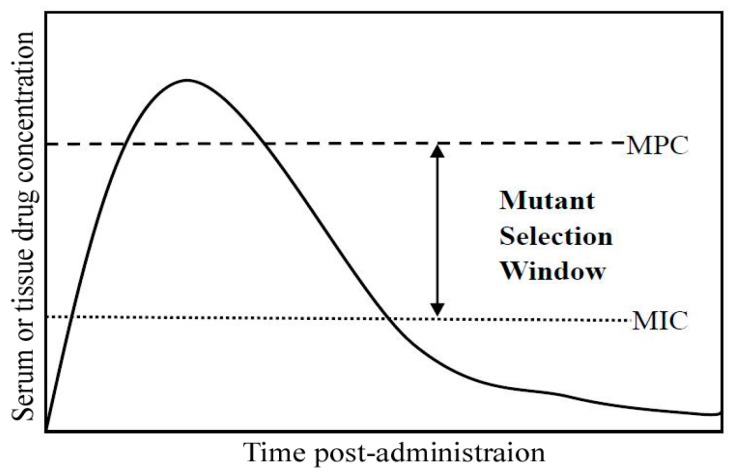
Pharmacodynamic characteristics of the mutant selection window. Drug concentrations indicated by the double-headed arrow between the mutant prevention concentration (MPC) and minimal inhibitory concentration (MIC) represent the mutant selection window.

According to the proposed hypothesis for the mutant selection window (MSW), the antibiotic concentration plays an important role in selecting or enriching resistant mutants. As shown in Figure 1, the MSW represents an antibiotic concentration that is above the MIC and below the mutant prevention concentration (MPC) [15,16]. If the antibiotic concentration is below the MIC, the number of selected or enriched resistant mutants is reduced, but the antibiotic fails to eradicate the pathogenic bacteria and this leads to treatment failure. If an antibiotic concentration is within the MSW, it has a therapeutic effect, but it can enhance selection or enrichment of resistant mutants. Moreover, the MPC of an antibiotic for bacteria indicates the concentration where no resistant colony is found, even when 10^10^ bacteria are plated. If the MNZ concentration is above the MPC, not only can a full therapeutic *H. pylori* effect be obtained, but it can also reduce the selected or enriched resistant mutants. Therefore, an alternative strategy for reducing the MPC and narrowing the MSW of MNZ for *H. pylori* is needed to overcome the increasing amount of resistant mutants. Human lysozyme (hLYS) is a type of antimicrobial substance that is secreted from various cells and tissues [17,18,19]. Moreover, hLYS has muramidase activity that can catalyze the hydrolysis of bacterial cell wall peptidoglycans, including β-1,4-linked *N*-acetylmuramic acid (MurNAc) and *N*-acetyl glucosamine (GlucNAc) [20]. Additionally, some reports found that another effect occurs via muramidase-independent processes instead of a cationic dependent mode [21,22]. These effects can increase the permeability of bacterial cells and favor the entry of antibiotics into bacterial cells. Indeed, in this study, we found that the application of hLYS could dramatically reduce the concentration and time of the MNZ bactericidal kinetics, and alter the dimensions of the MSW by lowering the MPC. These findings indicate that hLYS has synergistic anti-*H. pylori* effects with MNZ and reduces the MPC, which is helps to reduce the incidence of resistance mutations.

## 2. Results

### 2.1. H. pylori Was Insensitive to Lysozyme

The ATCC43504 strain of *H. pylori* was used to examine the sensitivity of lysozyme with 0.3 or 30 mg/mL of hLYS. Approximately 7.5 × 10^7^ cells/mL were treated with hLYS, and cell survival of *H. pylori* was determined after 0, 2, 4, or 6 h. We found that *H. pylori* was highly resistant to hLYS (Table 1). This finding was consistent with those of a previous study [23]. The gastric emptying time was about 4–6 h, and the concentration of hLYS in the stomach would decline with gastric emptying. Therefore, it was difficult to clear *H. pylori* with oral hLYS alone.

### 2.2. hLYS Promotes Increased Outer Membrane Penetration

**Figure 2:**
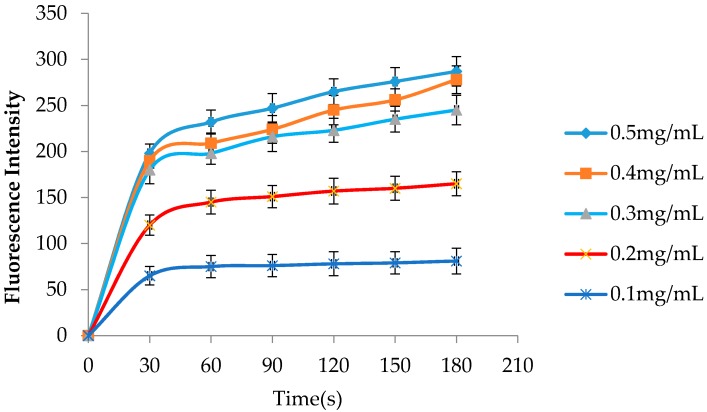
Effects of hLYS on permeability of the *H. pylori* outer membrane. The hLYS with different concentrations detect its ability of increasing membrane permeability through the change of the fluorescence intensity with time.

NPN has been used a hydrophobic fluorescent probe to investigate interactions between substances and the outer membrane of bacteria and to detect the permeabilization of the outer membrane [24,25]. The fluorescence of NPN is weaker in an aqueous solution and can become stronger after penetrating into the outer membrane hydrophobic environment. As shown in Figure 2, the fluorescence intensity was obviously enhanced when hLYS was added to the *H. pylori* suspension. These findings showed that penetration of the *H. pylori* outer membrane could be promoted by hLYS in a dose-dependent manner.

### 2.3. hLYS Can Disrupt the Integrity of the Inner Membrane

**Figure 3:**
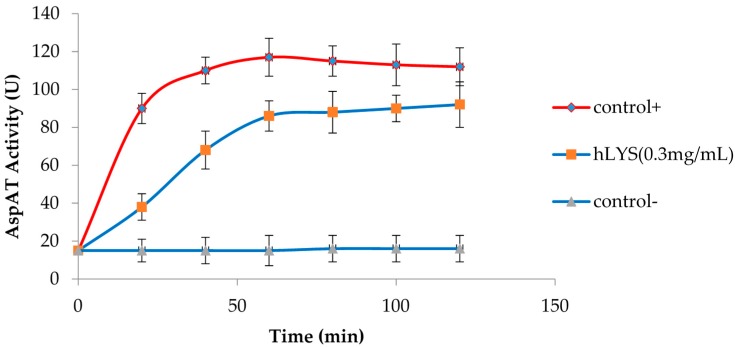
Increased AspAT activity resulting from the release of AspAT from *H. pylori* treated with hLYS. Triton (0.2%) was used as a positive control; 0.9% NaCl solution was used as a negative control.

To further probe the effects of hLYS on the integrity of the *H. pylori* inner membrane, an extracellular AspAT activity assay was used. Extracellular AspAT activity could increase as a consequence of enzyme release from the cytoplasm when the *H. pylori* inner membrane becomes damaged or destabilized by hLYS. The finding also showed that hLYS could induce changes in the *H. pylori* inner membrane integrity and promote the permeability of AspAT, which was indicated by a marked reduction in absorbance at 340 nm when NADH was oxidized to NAD+, and a marked increase in extracellular AspAT activity resulting from aspartate aminotransferase release from the cytoplasm (Figure 3).

### 2.4. hLYS Could Promote PI Uptake by Cells

**Figure 4:**
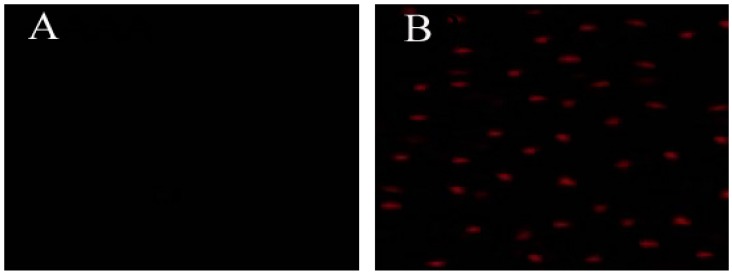
Effect of hLYS on *H. pylori* membrane permeabilization. *H. pylori* was incubated with (**A**) PBS (a control) or (**B**) 50 μM PI and 0.3 mg/mL hLYS for 90 min at room temperature under microaerophilic conditions. The cells were observed using fluorescence confocal microscopy. (**A**) No fluorescence was observed, indicating that PI could not enter the cells; (**B**) A few red fluorescence dots could be observed, demonstrating that PI could enter cells and bind to DNA.

To further clarify whether hLYS could promote cell absorption of external substances under conditions of increasing penetration of the outer membrane and disrupting the integrity of the inner membrane, we used a visual method. PI was absorbed into cells and bound to DNA, resulting in the emission of red fluorescence that could be easily detected using fluorescence confocal microscopy [26]. As shown in Figure 4, this experiment demonstrated that hLYS could promote PI uptake into cells, which could be visualized. If lysozyme could promote PI absorption, this effect would also promote the absorption of metronidazole into cells by increasing membrane permeability caused by membrane damage induced by hLYS. The concentration of metronidazole in the cells could be increased, resulting in enhanced antimicrobial activity.

### 2.5. Bactericidal Kinetics of hLYS and Metronidazole Treatment

**Figure 5:**
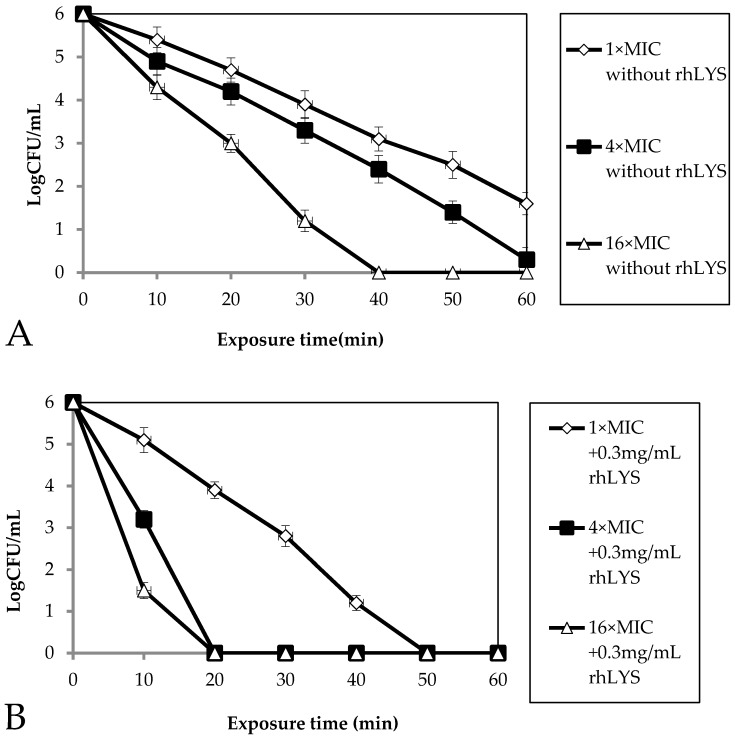
Bactericidal kinetics. The bactericidal kinetics of metronidazole without hLYS (**A**) and of metronidazole with 0.3 mg/mL hLYS (**B**) against *H. pylori* were monitored at 10 min intervals for 1 h at 37 °C. Subsequently, aliquots were diluted (serial 10-fold dilutions) and plated for CFU counts after 72 h incubation at 37 °C.

*H. pylori* cells are highly resistant to the bactericidal effects of lysozyme, as they can survive for hours in up to 30 mg/mL (our study) or 50 mg/mL (another study [27]) lysozyme, which indicated that oral lysozyme alone may not achieve the maximal effect of killing *H. pylori*, but 0.3 mg/mL hLYS can enhance cell membrane permeability. The bactericidal activity kinetics against *H. pylori* ATCC43504 were investigated by counting the number of surviving bacteria according to a previously reported method [27]. Cell survival was measured within the first 60 min after bacterial exposure to different concentrations of metronidazole or to metronidazole along with 0.3 mg/mL hLYS at different time points. As shown in Figure 5, 0.3 mg/mL hLYS synergized with metronidazole to kill *H. pylori*. Furthermore, regarding the bactericidal kinetics, the concentration of metronidazole become markedly lower and bactericidal time become shorter, so while metronidazole cannot completely kill *H. pylori* within 60 min at a concentration of 4× MIC, it could completely kill *H. pylori* within 20 min at a concentration of 4× MIC.

### 2.6. Effect of hLYS on the MPC and MIC of Metronidazole to H. pylori

We found that hLYS could significantly reduce the MIC and MPC of metronidazole on *H. pylori* using an agar dilution method (Table 2). The MIC and MPC of metronidazole in *H. pylori* changed from 8 and 153.6 μg/mL to 1 and 25.6 μg/mL, respectively. The MSW of metronidazole in *H. pylori* narrowed from 145.6 to 24.6 μg/mL. Considering the theoretical basis of the MSW, this effect should reduce the probability of mutations arising that render *H. pylori* resistant to antibiotics.

## 3. Discussion

It has been reported that resistance to MNZ accounts for a reduction in efficacy of up to 50% for bismuth- and PPI-containing triple therapies [11]. Recently, studies have shown that *H. pylori* resistance to MNZ reaches 17%–44% in Europe and America, while the highest resistance rates have been estimated to be 80%–92.4% in Africa [28,29]. In the last decade, the rate of *H. pylori* resistance to MNZ has significantly increased in China from 23.8% to 56.6% [30]. To achieve the therapeutic effect of *H. pylori* resistance to MNZ, some improved therapeutic methods have been achieved by increasing the MNZ dose (1600 mg/day or even 2 g/day) and prolonging the duration of therapy (from the original 7 days to the present 10 or 14 days) [12,31,32,33]. Although these methods have achieved a certain therapeutic effect, according to the MSW theory, the status of *H. pylori* resistance to MNZ will worsen using the above treatment strategy because the concentration of MNZ is between the MIC and MPC, and a prolonged treatment time can enrich and promote *H. pylori* resistance mutations to MNZ [13,14,15,16]. If the concentrations of MNZ are above the MPC, *H. pylori* resistance mutations rarely emerge, but an MNZ concentration that remains above the MPC in the stomach is difficult to achieve because of the stomach emptying. MNZ resistance have been reported to be associated with mutations of oxygen independent NADPH nitroreductase (RdxA) and NADH flavin oxidoreductase (FrxA) [34,35]. The study confirmed that the sensitive strain of MNZ become resistant strains due to rdxA and frxA gene mutation using natural transformation experiments through the selection of low concentration of MNZ [36]. Therefore, a method of lowering the MPC, narrowing MSW and enhancing the bactericidal dynamics, i.e., to shorten the bacterial killing time, must be found to block the resistance mutations induced by MNZ and enhance its bactericidal effects. In this study, a method to increase the permeability of *H. pylori* was used that would be beneficial for MNZ via effects on the bacterial cell wall and cell membrane, which could lower the MPC of MNZ and result in more rapid killing of *H. pylori*. Although MNZ only remained for a short time in the stomach, a more effective inhibition and killing of *H. pylori* could be achieved, and the bacteria would be less likely to develop drug resistance to MNZ. Previous studies established that *H. pylori* has a high tolerance to lysozyme, as *H. pylori* could not be completely killed at a concentration of 50 mg/mL [23]. Indeed, in this study, we found that *H. pylori* could not be completely killed by 30 mg/mL hLYS for 6 h, while 0.3 mg/mL hLYS demonstrated weaker activity towards *H. pylori*, as many *H. pylori* survived after hLYS acted on the bacteria for 6 h. Therefore, a complete cure of *H. pylori* infection could not be attained by direct oral administration of lysozyme. However, lysozyme can act on the bacterial cell wall peptidoglycans β-1,4-linked *N*-acetylmuramic acid (MurNAc) and *N*-acetylglucosamine (GlucNAc), and it has the characteristic of a cation, which can increase cell permeability, which is beneficial for antibiotic entry into the cell and can enhance antibacterial activity. In the present study, we found that 0.3 mg/mL recombinant human lysozyme increased the permeability of the cell wall and destroyed the integrity of the cell membrane of *H. pylori* (Figure 1 and Figure 2). Additionally, it also enhanced the absorption of outside material into cells (Figure 3). We carried out further assessments and found that 0.3 mg /mL hLYS was synergistic with MNZ against *H. pylori*; it increased the MNZ bactericidal kinetics for a given concentration, lowering the bactericidal time. Thus, the conditions changed from the original observation that MNZ could not completely kill *H. pylori* at a concentration of 4× MIC in 60 min, to a state where it could completely kill *H. pylori* at a concentration of 4× MIC in 20 min (Figure 5). We also found that 0.3 mg/mL hLYS could significantly reduce the MPC of MNZ (Table 2). Our findings indicated that a concentration above the MPC could easily be reached in the stomach using the original oral dose for MNZ and hLYS to treat *H. pylori* infection. This treatment would not only enhance the treatment effects of MNZ but also greatly reduce the resistance of *H. pylori* to MNZ. This study may provide a new technique to solve the problem of antibiotic resistance for the treatment of bacterial infections in the clinic.

## 4. Materials and Methods

### 4.1. Bacterial Strain and Chemicals

The bacterial strain ATCC43504 of *H. pylori* was used in this study and was maintained in our laboratory. hLYS was purchased from Prospec-Tany TechnoGene. Ltd. (Rehovot, Israel). The compounds metronidazole, *N*-2-hydroxyethylpiperazine-*N*′-2-ethanesulfonic acid (HEPES), malate dehydrogenase (MDH), pyridoxal-5′-phosphate l-aspartate, NADH, α-ketoglutarate, NPN and propidium iodide (PI) were purchased from Sigma Chemical Co. (St. Louis, MO, USA).

### 4.2. Culture Conditions for H. pylori

*H. pylori* was cultured on Columbia blood agar plates (CM0331B; Oxoid, Basingstoke, UK) supplemented with 10% defibrinated sheep blood. Plates were incubated at 37 °C for 3 days in microaerophilic atmospheric conditions that were established using a microaerophilic gas-generating bag (QingDao Hope Bio-Technology Co., Ltd., QingDao, China).

### 4.3. Cell Survival Assays to Determine hLYS Sensitivity

*H. pylori* strains were grown on Columbia blood agar plates supplemented with 10% defibered sheep blood for 24 h until they reached log phase. The cells were centrifuged at 5000× *g* for 10 min, and then, the cells were suspended in phosphate-buffered saline (PBS) and adjusted to a concentration of 10^8^ CFU/mL. A final concentration of 0.3 or 30 mg/mL of lysozyme was added to a final 7.5 × 10^7^ CFU/mL of *H. pylori* with full mixing. The cell suspensions were incubated at 37 °C under microaerophilic atmosphere conditions with shaking at 200 rpm. Samples were then harvested at various time points (0, 2, 4, and 6 h), and plated onto Columbia blood agar plates after serial dilution. Colony counts were carried out after 3 days of incubation in a microaerobic atmosphere at 37 °C.

### 4.4. Permeabilization Assay of the Outer Membrane (OM)

The effect of *H. pylori* OM permeabilization on hLYS was determined using a fluorescent probe of hydrophobic NPN [24,25]. A 50 mL sample of log phase *H. pylori* was centrifuged at 5000× *g* for 10 min. Cells were collected, washed three times, and suspended in 5 mM sodium HEPES buffer (pH 7.2). Then, the optical density at 600 nm was adjusted to 0.5. Samples (4 mL) containing 2 mL *H. pylori* suspension, 1 mL hLYS at 0.1, 0.2, 0.3, 0.4, or 0.5 mg/mL and 1 mL of 40 mM NPN were prepared. The control was 2 mL of 5 mM HEPES without cells. Excitation and emission wavelengths for NPN were set at 350 and 420 nm, respectively. Increases in fluorescent intensity resulting from NPN penetrating into the OM were measured at 30 s intervals until no further increase was observed (F4600 fluorescence spectrophotometer, Hitachi, Tokyo, Japan). The experiments were carried out three times to obtain the average value.

### 4.5. Integrity Assay of the Inner Membrane (IM)

The effect of hLYS on *H. pylori* IM integrity was determined by measuring the release of aspartate aminotransferase (AspAT) from *H. pylori* into the culture medium. AspAT activity was indicated by the amount of aspartate converted to oxaloacetate by measuring the malate dehydrogenase (MDH)-catalyzed, NADH-dependent conversion of oxaloacetate to malate. The enzymatic activity of AspAT was detected by the reduction in absorbance at 340 nm. A 50 mL sample of log phase *H. pylori* was centrifuged at 5000× *g* for 10 min. The cells were collected, washed three times, and suspended in a 0.9% NaCl solution, after which the optical density at 600 nm was adjusted to 1.0 in accordance with a previously reported method with slight modifications [37]. The AspAT reaction mixture (4 mL) contained 2 mL *H. pylori* suspension, 1 mL hLYS at 0.3 mg/mL, 20 mM Tris (pH 7.8), 50 µM pyridoxal-5′-phosphate, 20 mM α-ketoglutarate, 50 mM l-aspartate, 0.2 mM NADH, and 12U MDH. Positive and negative controls were 0.2% Triton and 0.9% NaCl, respectively. An increase in AspAT activity was represented by the reduction in absorbance at 340 nm using a spectrophotometer. One unit of AspAT was defined as the amount of enzyme that catalyzed the oxidation of 1 µmol NADH min^−1^ and led to a reduction in absorbance of 0.001 min^−1^. AspAT activity was measured at 20 min intervals until no further increase resulting from the increasing penetration of AspAT from cells by hLYS was detected. The experiments were carried out three times to obtain the average value.

### 4.6. Cell Uptake Assay

A mixture of 1 mL of *H. pylori* suspension (optical density 0.5 at 600 nm) and 1 mL of 30 mg/mL hLYS or 1 mL of PBS buffer (pH = 7.2, as a control) were incubated for 2 h at 37 °C, then 2 mL of 100 μM propidium iodide (PI) was added. The mixture was further incubated for 5 min at room temperature in the dark. PI entered the cell, bound to DNA and emitted red fluorescence only under conditions that caused damage to the cell membrane. Red fluorescence could be detected with a fluorescence confocal microscope (LSM 710 Meta, Zeiss, Jene, Gemany). The experiments were carried out three times to obtain the average value.

### 4.7. Bactericidal Kinetics Activity Based on hLYS and Metronidazole Synergy

The MIC was tested using a standard serial dilution method that was previously described by our group [27]. Briefly, *H. pylori* was cultured overnight in BHI broth under microaerophilic conditions at 37 °C, and then the mixture was centrifuged to collect the bacterial pellet at 5000× *g* for 10 min. The pellet was properly diluted and its optical density at 600 nm (OD_600_) was adjusted to 1.0. The number of *H. pylori* cells corresponded to approximately 1 × 10^8^ colony forming units (CFU)/mL. MNZ was serially diluted, and the concentration range of MNZ was 1 to 512 μg/mL. Additionally, 10 μL of bacterial suspension containing 1 × 10^6^ CFU of bacteria was added to each 1 mL serial diluted solution. After 36 h of culture with shaking at 200 rpm under microaerobic conditions at 37 °C, the MIC was taken as the lowest drug concentration at which the tube was optically clear without any visible turbidity or growth. The experiments described above were conducted in triplicate. The bactericidal kinetics assay of hLYS plus MNZ against *H. pylori* was carried out as our group reported previously with some modifications [27]. Aliquots (1 mL) of different concentrations of MNZ culture media were prepared to obtain final concentrations of 1× MIC, 4× MIC, or 16× MIC containing 10^6^ CFU/mL *H. pylori* (at the exponential phase of growth) and 0.3 mg/mL hLYS; the control had no hLYS. After 0, 10, 20, 30, 40, 50 and 60 min of incubation under microaerophilic conditions at 37 °C, the cultures were serially diluted by adding 20 μL of culture to 180 μL of BHI broth. The samples were fully mixed and 50 μL of each dilution was plated on Columbia blood agar (CM0331B, Oxoid) supplemented with 7% defibered sheep blood for CFU counts after 48 h of incubation under microaerophilic conditions at 37 °C. The above experiments were conducted in triplicate.

### 4.8. Measurement of MPC

MPC measurements were performed as previously described with some modifications [38,39]. Briefly, *H. pylori* was cultured in BHI broth containing 7% fetal bovine serum and incubated for 24 h. Cultures were centrifuged at 5000× *g* for 10 min and resuspended in BHI broth to obtain a concentration of 10^11^ CFU/mL. A series of Columbia blood agar plates supplemented with 7% defibered sheep blood, various known MNZ concentrations and 0.3 mg/mL hLYS were plated with 100 μL of a suspension containing 10^10^ CFU of *H. pylori* and compared with MNZ alone. The inoculated plates were incubated for 72 h under microaerophilic conditions at 37 °C and screened visually to count the CFU. The lowest concentration of drug without visual colony growth was set as a provisional mutation prevention concentration (MPCpr) after culture for 72 h. After repeating the above operation, MPCpr was set as the baseline with a 20% linear decreasing concentration of antibiotic, and agar plates containing different concentrations of drug were prepared. The MPC was defined as the lowest concentration of drug that still did not support visual colony growth after 72 h of culture. The experiments were carried out three times.

### 4.9. Statistical Analysis

The difference was statistically analyzed between the MNZ-treated bacterial samples and MNZ + hLYS-treated bacterial samples were statistically analyzed using one-way analysis variance and Dunnett’s multiple comparison test. The significant differences between different bacterial samples were defined as *p* < 0.05.

## 5. Conclusions

MNZ is an effective agent used to eradicate *H. pylori*. The emergence of widespread *H. pylori* resistance to MNZ has affected its therapeutic effects. Effective treatments to overcome MNZ resistance can be obtained by increasing the MNZ dose and prolonging the duration of therapy. According to the MSW theory, resistant mutants would thus be selected or enriched. A strategy to enhance the bactericidal dynamics and lower the resistant mutant prevention concentration would overcome this problem. hLYS possesses muramidase activity that can catalyze the hydrolysis of bacterial cell wall peptidoglycans, including β-1,4-linked *N*-acetylmuramic acid (MurNAc) and *N*-acetylglucosamine (GlucNAc). Additionally, hLYS can also act in a cationic-dependent mode. These effects can increase the permeability of bacterial cells. In this study, our findings demonstrated that hLYS indeed could promote increased penetration of the outer membrane, cause disruption of the integrity of the inner membrane, and promote the uptake of extracellular substances into cells. Further studies found that the bactericidal activity kinetics of MNZ were markedly enhanced from the original in which MNZ could not completely kill *H. pylori* at a concentration of 4× MIC in 60 min to a state where it could completely kill *H. pylori* at a concentration of 4× MIC in 20 min. The MIC and MPC for MNZ in *H. pylori* were obviously reduced from 8 and 153.6 μg/mL to 4 and 25.6 μg/mL, respectively. The MSW was significantly narrowed from 145.6 to 21.6 μg/mL. These findings demonstrated that hLYS with MNZ produced a very low MIC, rapidly killed bacteria, and had a low propensity for generating resistance because of the narrow MSW. In conclusion, the present study establishes that hLYS acts in combination with MNZ to eradicate *H. pylori* infections and reduce the rate of resistance mutations by increasing cell permeability.

## Figures and Tables

**Table 1:** Sensitivity of *H. pylori* to rhLYS.

Time (h)	Cell Survival			
	0.3 mg/mL rhLYS		30 mg/mL hLYS	
	CFU/mL	% Survival	CFU/mL	% Survival
0	7.5 × 10^7^	100%	7.5 × 10^7^	100%
2	5.3 × 10^7^	70.6%	1.6 × 10^7^	21.3%
4	1.2 × 10^7^	16%	4.8 × 10^6^	6.4%
6	8.7 × 10^6^	11.5%	5.3 × 10^4^	0.07%

*H. pylori* were treated with 0.3 or 30 mg/mL hLYS for 0, 2, 4 or 6 h. The number of surviving *H. pylori* cells was determined by cell colony counting.

**Table 2:** Effect of hLYS (0.3 mg/mL) on the MIC, MPC and MSW of MNZ to *H. pylori*.

Formulation	MIC	MPC	MSW
MNZ	8 μg/mL	153.6 μg/mL	145.6 μg/mL
MNZ + hLYS	1 μg/mL *	25.6 μg/mL **	24.6 μg/mL **

** *p* < 0.01 compared with MNZ; * *p* < 0.05 compared with MNZ.
